# A real-world experience of venetoclax combined with hypomethylating agents vs. monotherapy hypomethylating agents in patients with myelodysplastic syndromes and chronic myelomonocytic leukemia patients

**DOI:** 10.3389/fphar.2024.1265840

**Published:** 2024-05-02

**Authors:** Ludan Zhang, Rui Ge, Deng Pan, Pengjie Yue, Jingwen Zhang, Renjie Bian, Xiaojing Yan

**Affiliations:** Department of Hematology, The First Affiliated Hospital of China Medical University, Shenyang, China

**Keywords:** myelodysplastic syndromes, chronic myelomonocytic leukemia, venetoclax, hypomethylating agents, real-world study

## Abstract

**Introduction:**

Current clinical research has reported the effectiveness and safety of venetoclax in combination with hypomethylating agents (VEN-HMA) in patients with myelodysplastic syndromes (MDS) and chronic myelomonocytic leukemia (CMML). Thus, this study aimed to examine the effectiveness and safety of VEN-HMA therapy in patients with MDS and CMML and compared its short-term and long-term therapeutic effects with HMA monotherapy.

**Method:**

We analyzed data from our center, comprising 19 patients with MDS and CMML who received VEN-HMA therapy, compared to 32 patients treated with HMA monotherapy.

**Results:**

The overall response rate (ORR) in the VEN-HMA group was 73.7%, compared to 59.4% in the HMA group. The survival analysis revealed that the median overall survival (mOS) time in the VEN-HMA group was 16 months, with a median progression-free survival (mPFS) time of 9 months, both of which were longer than those observed in the HMA group (*p* < 0.05). Key adverse events (AEs) included grade 3–4 neutropenia (89.5% in VEN-HMA group vs. 87.5% in HMA group), grade 3–4 thrombocytopenia (73.7% vs. 71.9%), and anemia (73.7% vs. 90.6%). Infection of grade 3 or higher occurred in 63.2% of patients in the VEN-HMA group and 65.6% of patients in the HMA group.

**Discussion:**

Our study has confirmed the effectiveness and safety of the combined treatment of HMAs and venetoclax, which offers significant advantages to patients due to the relatively high and rapid response rates.

## 1 Introduction

Myelodysplastic syndromes (MDS) are clonal myeloid neoplasms characterized by refractory hemocytopenia and morphologic hematopoietic pathology, with a high risk of progression to acute myelocytic leukemia (AML) (Sekeres and Taylor, 2022). Currently, clinical diagnosis mainly adopts the 2016 revision to the World Health Organization (WHO) classification of myeloid neoplasms and acute leukemia, which is based on identified genetic and morphological abnormalities, emphasizing the importance of genetics in defining the disease (Arber et al., 2016). The annual incidence of MDS is approximately four per 100,000 people, predominantly affecting middle-aged and older individuals, with a higher prevalence in men than women (Rollison et al., 2008). The common clinical prognostic stratification system for MDS is the Revised International Prognostic Scoring System (IPSS-R), which incorporates cytogenetics, bone marrow blasts, hemoglobin, platelets, and absolute neutrophil counts to predict the clinical outcomes of untreated MDS patients (Greenberg et al., 2012).

Chronic myelomonocytic leukemia (CMML) is a rare clonal hematopoietic malignancy characterized by clinical features of both myelodysplasia and myeloproliferation, which also progresses to AML with a high risk of mortality (Patnaik and Tefferi, 2022). The clinical manifestations of CMML are highly diverse and characterized by persistent peripheral blood mononucleosis, as well as the development and proliferation of one or more blood cell lineages (Arber et al., 2016). The overall prognosis of CMML is generally poor, with a median OS time of 17 months (Guru Murthy et al., 2017). Prognosis is stratified according to the CMML-specific Prognostic Scoring System (CPSS) (Tremblay et al., 2021).

According to the 2023 NCCN guidelines, hypomethylating agents (HMAs) are recommended for lower-risk MDS patients with clinically relevant thrombocytopenia or neutropenia, as well as higher-risk patients who are ineligible for allogeneic hematopoietic stem cell transplantation (allo-HSCT) (Greenberg et al., 2022). To date, there is a lack of standardized CMML treatment strategies that can significantly prolong patient prognosis. Thus, CMML therapy primarily mirrors that of MDS. Patients with low-risk CMML should undergo close clinical monitoring and receive supportive care. Patients with high-risk CMML presenting obvious symptoms should be monitored and given cytotoxic therapy, HMAs, or allo-HSCT. Notably, HMAs are the sole agents approved by the U.S. Food and Drug Administration (FDA) for CMML treatment (Tremblay et al., 2021).

Venetoclax is a novel oral BCL-2 inhibitor that primarily kills tumor cells by inducing endogenous apoptotic pathways. In November 2018, the FDA expedited the approval of venetoclax in combination with HMAs for treating AML. Given the high risk of transformation to AML, several clinical trials have explored the effectiveness of VEN-HMA in patients with MDS, yielding promising therapeutic outcomes (Ball et al., 2020). Recently, a phase 1-2 trial conducted at a single center confirmed the effectiveness and safety of azacitidine plus venetoclax in patients with high-risk MDS or CMML (Bazinet et al., 2022). However, real-world evidence on this combination remains limited. Thus, this study aimed to examine the effectiveness and safety of VEN-HMA therapy in patients with MDS and CMML and compare its short-term and long-term therapeutic effects with HMA monotherapy.

## 2 Methods

### 2.1 Study design and patients

We retrospectively analyzed 51 patients with MDS or CMML who received either VEN-HMA therapy or HMA monotherapy in our institution. The diagnostic criteria followed the 2016 revision of the WHO Classification of Hematolymphoid Tumors. Each patient received at least one administration of either the combination or monotherapy between May 2019 and February 2023 and experienced at least one evaluation after therapy. Inclusion criteria comprised patients with secondary MDS or those exposed to HMAs, while patients who progressed or died within one treatment cycle were excluded. Survey data were collected from electronic medical records. Complete blood counts, blood biochemical items, electrocardiograms, and CT scans of the lungs were conducted before the first treatment. Additionally, baseline assessments included morphology, immunophenotype, cytogenetics, and next-generation sequencing (NGS) of bone marrow. Patients were stratified into lower- or higher-risk groups based on IPSS-R and CPSS criteria, and all patients provided informed consent.

### 2.2 Treatment and response criteria

Patients with MDS or CMML who were newly diagnosed or those with treatment failure received azacitidine (75 mg· m^−2·^ D^−1^, 7 days) or decitabine (20 mg· m^−2^· D^−1^, 5 days) either with or without venetoclax (100 mg, day 1; 200 mg, day 2; 400 mg, days 3–14). Three patients were treated with venetoclax at reduced doses due to prolonged myelosuppression. Certain patients underwent bone marrow examination post-treatment. The last follow-up was conducted in May 2023. The failure treatment regimens before venetoclax included D-CAG and single HMA therapy.

Given the absence of international consensus criteria for CMML, researchers typically refer to the adult MDS/MPN International Working Group (IWG) 2006 criteria. Complete response (CR) is defined as bone marrow myeloblasts <5% and full recovery in peripheral blood. Marrow CR (MCR) refers to bone marrow myeloblasts less than 5% and a 50% decrease from pretreatment. Hematologic improvement (HI) indicates specific responses of three peripheral blood lineages. Partial response (PR) meets the criteria of CR but shows a reduction in bone marrow blast cells <50%, still exceeding 5%. Stable disease (SD) is defined as not meeting the minimum criteria for a PR, with no evidence of progression for at least 8 weeks. Treatment failure (TF) refers to the progression of disease or death during treatment, while progressive disease (PD) includes scenarios where bone marrow myeloblasts increase to >50% or meet specific criteria: neutrophil granulocyte, platelet decrease (>50%), hemoglobin decrease (>20 g/L), or transfusion dependence (Cheson, 2006).

The ORR encompasses the total rates of CR, MCR, HI, and PR patients. The median duration of response (mDOR) measures the time from when 50% of patients first achieved CR or PR to disease progression. OS refers to the time from the first venetoclax dose to death from any cause. Given the challenges in reaching OS endpoints, requiring longer follow-up, PFS was also selected as a study endpoint, referring to the time from the first venetoclax dose to disease progression or death.

All AEs of the patients in our study were assessed using Common Terminology Criteria for Adverse Events 5.0 (CTCAE), which was categorized into grades 1 to 5. Any AEs occurring after venetoclax treatment were documented as drug-related.

### 2.3 Statistics

Statistical analyses were conducted with Statistical Product and Service Solution (SPSS) 20.0 and GraphPad Prism 8. The chi-squared tests and non-parametric test (Mann–Whitney test) were used for categorical and continuous variable comparison. A Kaplan–Meier curve was used to describe the survival characteristics by estimating OS and PFS. The statistical difference was compared using the log-rank test. The hazard ratios (HRs) and 95% confidence interval (CIs) were recorded. All statistical tests were two-sided, and a value of *p* < 0.05 was considered statistically significant.

## 3 Results

The basic characteristics of patients, evenly distributed between the two study groups, are shown in Table 1. A total of 51 patients completed the study, with 19 in the VEN-HMA group and 32 in the HMA group. In the VEN-HMA group, there were 11 patients with MDS, while the remainder had CMML. Conversely, the HMA group consisted of 20 patients with MDS and 12 with CMML. The median follow-up times were 16 and 24 months for VEN-HMA and HMA, respectively. As seen in Table 1, four patients in the VEN-HMA group underwent allo-HSCT, while none in the HMA group received transplantation (*p* = 0.007). A total of 41 patients underwent NGS before treatment. Table 1 lists the genes with mutation frequencies >10%. The two most common genes in the VEN-HMA group were ASXL1 and U2AF1, while in the HMA group, they were U2AF1 and SETBP1. However, no statistical significance was observed between the two groups for any of the genes.

**TABLE 1:** Basic characteristics of patients.

	N (%) or median (range)		
Variables	Ven-HMA (n = 19)	HMA (n = 32)	*P*
Age, years	57 (32–76)	61 (28–86)	0.726
Male, sex	12 (63.2%)	26 (81.2%)	0.271
Disease type			0.745
MDS	8 (42.1)	12 (37.5)	
CMML	11 (57.9)	20 (62.5)	
ECOG PS before treatment			0.647
0	2 (10.5)	3 (9.4)	
1	10 (52.6)	16 (50.0)	
2	6 (31.6)	13 (40.6)	
3	1 (5.3)	0 (0.0)	
Bone marrow blast (%)^a^	7.20 (1.20–18.00)	7.70 (1.30–18.20)	1.000
Peripheral blood count^a^			
WBC, ×10^9^/L	4.32 (0.77–134.04)	3.49 (0.95–219)	0.628
Hemoglobin, g/L	61.5 (37–112)	74 (35–136)	0.312
Platelets, ×10^9^/L	53.5 (3–537)	75 (5–1,153)	0.801
Risk stratification^b^			0.980
Lower-risk	6 (31.6)	10 (31.2)	
Higher-risk	13 (68.4)	22 (68.8)	
Cycles of therapy			0.662
<2	6 (31.6)	7 (21.9)	
≥2	13 (68.4)	25 (78.1)	
HMA history, n	8 (42.1)	0	
Combination medication			0.082
Azacitidine	16 (84.2)	18 (56.2)	
Decitabine	3 (15.8)	14 (43.8)	
Allo-HSCT, n	4 (21.1)	0 (0)	0.007
Mutations, n^c^			
ASXL1	7	5	0.288
U2AF1	4	8	0.740
SETBP1	1	6	0.237
RUNX1	4	2	0.364
TET2	1	4	0.579
TP53	4	1	0.167
NRAS	4	1	0.167

Abbreviations: MDS, myelodysplastic syndromes; CMML, chronic myelomonocytic leukemia; WBC, white blood cell; ECOG PS, Eastern Cooperative Oncology Group Performance Status.

^b^ The lower-risk group includes the IPSS-R (very low-risk group), the Low-Risk Group and Moderate Risk Group (≤3.5 points), and the CPSS: Low Risk and Moderate Risk group 1.

The higher-risk group includes the IPSS-R: Moderate Risk Group (>3.5 points), the High-Risk Group and Very High-Risk Group, and the CPSS: Moderate Risk 2 and High-Risk Groups.

^a^ One patient’s initial material was missing.

^c^ The genes with mutation frequencies ≥10% are listed in the table. Ten patients did not have NGS examinations.

**FIGURE 1:**
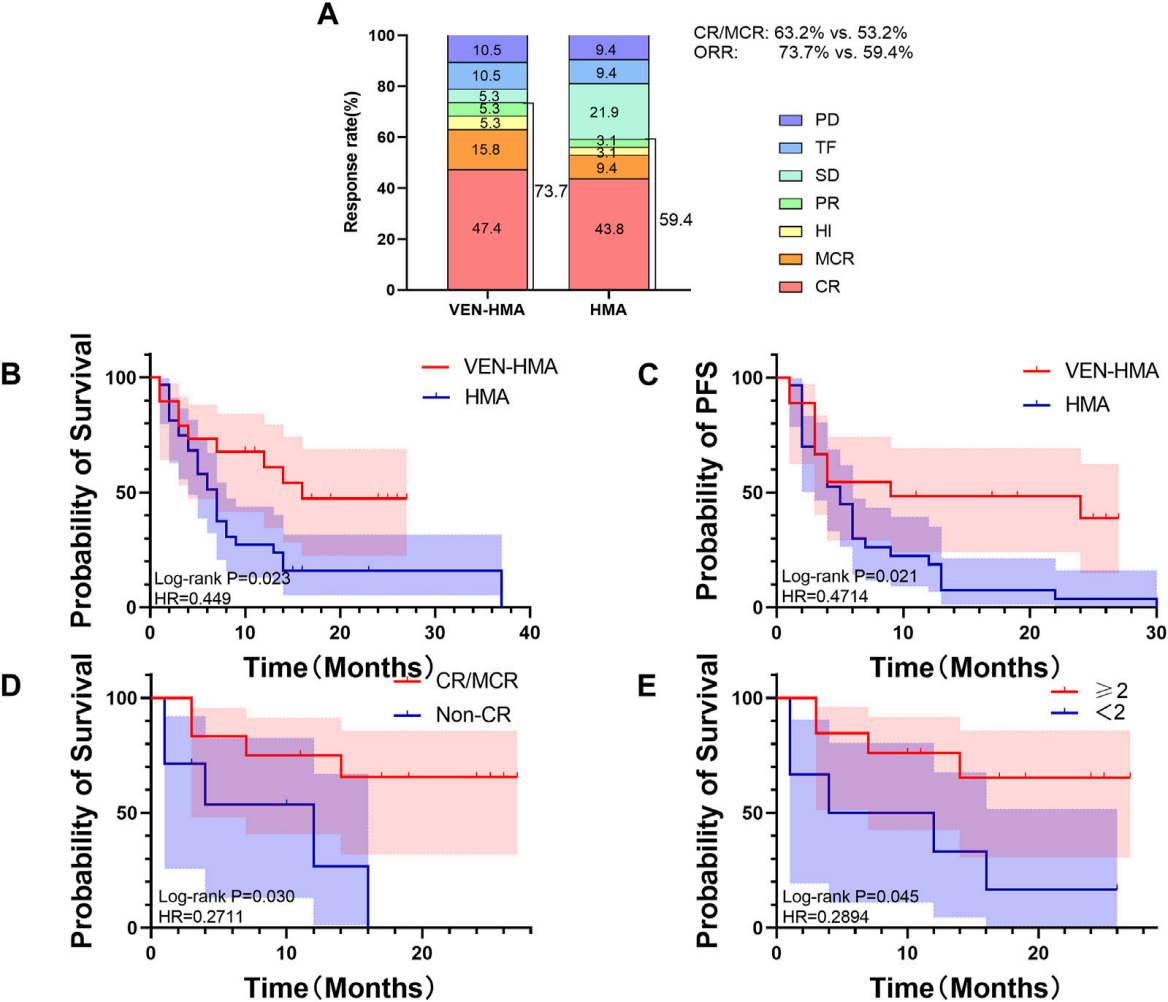
**(A)** Best treatment response rates in the VEN-HMA and HMA groups. CR, complete remission; MCR, marrow CR; HI, hematologic improvement; PR, partial response; SD, stable disease; TF, treatment failure; PD, progressive disease. **(B)** Overall survival in the VEN-HMA and HMA groups. **(C)** Progression-free survival in the VEN-HMA and HMA groups. **(D)** Comparison of overall survival between CR/MCR patients and non-CR patients in the VEN-HMA group. **(E)** Comparison of overall survival between patients who received more than two cycles in the VEN-HMA group.

Figure 1A presents the overview of treatment response on the basis of the IWG 2006 criteria. The ORR was 73.7% in the VEN-HMA group and 59.4% in the HMA group (*p* = 0.301). The treatment responses in the VEN-HMA group included CR (9/19), MCR (3/19), HI (1/19), PR (1/19), SD (1/19), TF (2/19), and PD (2/19). In the HMA group, these responses included CR (14/32), MCR (3/32), HI (1/32), PR (1/32), SD (7/32), TF (3/32), and PD (3/32).

Figures 1B and 1C display the survival data of the VEN-HMA and HMA groups. The mOS was significantly longer for patients treated with VEN-HMA than with HMA monotherapy (16 months vs. 7 months, *p* = 0.023). The PFS time between the two groups also showed significant differences (*p* = 0.021). In the VEN-HMA group, we performed a separate survival analysis focused on the treatment response and treatment cycle. Figure 1D shows that patients who achieved CR or MCR survived significantly longer than those who did not (*p* = 0.030). Patients who received more cycles of venetoclax had a longer OS than those who received <2 cycles of venetoclax (*p* = 0.045).

Any AEs observed during VEN-HMA therapy or HMA monotherapy are listed in Table 2. Grades 3–4 hematological AEs were frequently observed. Between the VEN-HMA group and the HMA group, the occurrence of grades 3–4 neutropenia, anemia, and thrombocytopenia was 89.5% vs. 87.5%, 73.7% vs. 90.6%, and 73.7% vs. 71.9%, respectively. In the VEN-HMA group, there were 4 and 2 cases of patients dying from hemorrhage and infection, respectively, during the treatment. In the HMA group, two patients and six patients died from hemorrhage and infection, respectively. Regarding non-hematological AEs, 12 patients (63.2%) and 21 patients (65.6%) in the two groups developed grade 3 or higher infections. The most common side effects of the data were neutropenia and anemia, with 17 cases and 18 cases, respectively. The top two ion disorders were hypocalcemia (63.2% vs. 56.3%) and hypokalemia (52.6% vs.18.8%). Several patients experienced abnormal liver function and kidney function, which were managed through symptomatic treatment, resulting in recovery from ion disorders and recovery of liver and kidney function.

**TABLE 2:** Adverse events.

	Ven-HMA (n = 19)				HMA (n = 32)			
	Grade 1–2	Grade 3	Grade 4	Grade 5	Grade 1–2	Grade 3	Grade 4	Grade 5
Leukopenia	0	4	11	0	3	8	18	0
Neutropenia	0	1	16	0	1	3	25	0
Anemia	4	12	2	0	3	22	7	0
Thrombocytopenia	1	1	13	0	1	5	18	2
Hemorrhage	0	0	0	4	0	0	1	2
Infection	1	8	2	2	1	13	2	6
Vomiting	1	0	0	0	2	0	0	0
Diarrhea	1	0	0	0	0	0	0	0
Arrhythmia	1	1	0	0	0	1	0	0
Heart failure	0	0	1	0	1	1	0	0
Oral mucositis	2	0	1	0	2	1	0	0
Rash	1	1	1	0	2	2	0	0
Fatigue	10	1	0	0	21	2	0	0
Hypercalcemia	1	0	0	0	0	0	0	0
Hypocalcemia	11	1	0	0	13	5	0	0
Hyperkalemia	1	1	0	0	1	1	0	0
Hypokalemia	7	3	0	0	1	4	1	0
Hyponatremia	4	1	0	0	3	2	0	0
Hypophosphatemia	5	1	0	0	7	2	0	0
Hyperbilirubinemia	3	0	0	0	1	5	1	0
Increased transaminase	5	0	0	0	8	3	4	0
Increased alkaline phosphatase	4	0	0	0	2	1	1	0
Increased creatinine	1	0	0	0	1	1	0	0

## 4 Discussion

Based on previous phase 1 results of azacitidine plus venetoclax in patients with high-risk MDS or CMML, the effectiveness and safety of this regimen were evaluated, showing an ORR of 87%, and venetoclax was well tolerated by patients with MDS and CMML (Bazinet et al., 2022). Our study was the first real-world research that retrospectively analyzed data collected from patients with MDS or CMML who received venetoclax and HMA treatment at our center, comparing the short-term and long-term effects with patients undergoing HMA monotherapy.

CMML is a relatively rare disease, with a median age at diagnosis of approximately 73–75 years, and patients are predominantly male (Patnaik and Tefferi, 2022). The epidemiology of MDS is consistent with patients with CMML, often occurring in older people (Li et al., 2022). In our study, the median age at diagnosis and sex ratio were consistent with previous studies. Most patients were in the higher-risk group, with 68.4% and 68.8% in the two groups, while other patients at lower risk due to symptomatic thrombocytopenia or agranulocytosis were treated with VEN-HMA treatment or HMA monotherapy. The three most common gene mutations in our study were ASXL1, U2AF1, and SETBP1. Numerous studies have shown that U2AF1 is associated with poor prognosis in MDS, especially in terms of OS (Wang et al., 2019; Wang et al., 2020). The presence of SETBP1 mutations and ASXL1 mutations usually indicates high white blood cell counts, extramedullary lesions, and poor prognosis (Patnaik et al., 2014).

The ORR of the VEN-HMA group was 73.7% vs. 59.4% in the HMA group in our study. Although the difference was not statistically significant, the ORR or CR/MCR rates of the VEN-HMA group were higher than those of the HMA monotherapy in our real-world data. Numerous clinical trial data results are shown below. One of the most important clinical trials in MDS, azacitidine-001, described that the mOS of the azacitidine group was 24.5 months, and the ORR was 29% (Fenaux et al., 2009). According to the CALGB 9221 trial, patients with low-risk MDS who were treated with azacitidine had an ORR of 59% and a median OS of 44 months (Silverman et al., 2006). Another clinical trial called SWOG S1117 compared the efficiency of azacitidine-based regimens and azacitidine monotherapy in patients with MDS and CMML, and the results showed the ORR of azacitidine was 38%, while the median OS was 15 months (Sekeres et al., 2017). Our study found the ORR of the VEN-HMA group was much higher than the clinical trial outcome of azacitidine monotherapy.

Comparing long-term curative effect, our results demonstrated that VEN-HMA therapy mOS and mPFS were significantly longer than HMA monotherapy. The mOS and mPFS in our VEN-HMA group were 16 months and 9 months, respectively, which aligns with the results observed in the phase 1 clinical trial (Bazinet et al., 2022). The OS in the VEN-HMA group did not exhibit an obvious advantage over the HMA monotherapy clinical trial described above. This could be due to the fact that most of the patients in our study were classified as high risk or very high risk. However, in cases where the basic information was similar, our VEN-HMA group showed a survival advantage over our HMA group. These results suggest that the combination of venetoclax may potentially extend the survival of patients with higher-risk MDS or CMML. Certainly, a larger sample and longer follow-up time would better support our results. In addition, we found that achieving CR/MCR and >2 cycles of venetoclax resulted in a significantly longer survival time in the VEN-HMA group. This finding highlights the importance of achieving CR at the earliest possible time and receiving an adequate number of treatment cycles to maximize the benefits for patients. However, it should be noted that a subset of patients who were within two cycles of VEN-HMA treatment had to discontinue the regimen due to disease progression or death. Unfortunately, these patients had a relatively short OS.

The most common grade 3–4 events that occurred in patients with MDS who received azacitidine were peripheral blood cytopenias, including neutropenia in 90% of patients, thrombocytopenia in 85% of patients, and anemia in 57% of patients (Fenaux et al., 2009). In the VEN-HMA group, nearly all patients experienced grade 3–4 hematological toxicity, neutropenia, and thrombocytopenia, which is consistent with the above study; however, the incidence of anemia was relatively low. Therefore, in terms of safety, AEs with VEN-HMA were predominantly myelosuppressive, with no obvious advantage over traditional therapy. All patients generally tolerated the treatment well, with most of the AEs resolving with symptomatic treatment. In comparison with the HMA group, the incidence of hematologic AEs and mortality were essentially the same and slightly higher in the non-hematologic AEs. A phase 3 study of patients with MDS treated with azacitidine indicated an incidence of grade 3–4 hematologic AEs that was basically consistent with our observations of both groups (Fenaux et al., 2009). A retrospective single-center study reported that the most common grade 3–4 AE was neutropenia (90%), with the most common non-hematologic AE being infection (60%) (Mei et al., 2023). Similarly, the results observed in our study were within this range.

Allo-HSCT is the only available cure for MDS (De Witte et al., 1990). Indications for allo-HSCT of MDS include patients aged <65 years in the higher-risk group or patients aged <65 years with severe hemopenia, failure of other treatments, or poor prognostic genetic abnormalities in the lower-risk group. For several reasons, <10% of patients with MDS accept allo-HSCT (Li et al., 2022). In our study, only four patients in the HMA-VEN group underwent allo-HSCT. In our center, most patients with MDS and CMML are unable to undergo all-HSCT due to old age, poor basic condition, or financial constraints.

Although few similar studies have been reported, our study has certain limitations. First, our findings were based on a single platform, and the sample size was relatively smaller. A larger sample size and data from multiple centers are needed to validate our findings. Second, our data may be affected by some confounding variables, but we did not give a multivariate adjustment because of the limited sample size. Third, our follow-up time is relatively short (median follow-up time: 20 months), and survival outcomes for some patients have not yet been observed.

Our findings have confirmed the effectiveness and safety of HMAs and venetoclax combination therapy. VEN-HMA therapy demonstrates an advantage over HMA monotherapy in treating patients with MDS or CMML. This combination therapy allows patients to achieve complete remission more rapidly, offering a promising new approach for patients. However, continuous exploration of the dosage and duration of this regimen is necessary to reduce the risk of AEs. We believe that more patients can benefit from and tolerate this therapy.

## Data Availability

The datasets presented in this article are not readily available because the data are not publicly available due to privacy or ethical restrictions. Requests to access the datasets should be directed to LZ, zhangludan1998@sina.com.
